# Electroclinical characteristics of photosensitive epilepsy: A retrospective study of 31 Chinese children and literature review

**DOI:** 10.3389/fped.2023.994817

**Published:** 2023-03-09

**Authors:** Bo Zhang, Tianyu Chen, Xiaosheng Hao, Meiying Xin, Jianmin Liang

**Affiliations:** ^1^ Department of Pediatric Neurology, First Hospital of Jilin University, Changchun, China; ^2^ Jilin Provincial Key Laboratory of Pediatric Neurology, Changchun, China; ^3^ Department of Pediatrics, The First Affiliated Hospital of Jinzhou Medical University, Jinzhou, China

**Keywords:** epilepsy, photosensitivity, children, electroencephalography, intermittent photic stimulation

## Abstract

**Objective:**

The objective of this study was to better understand the clinical features of photosensitive epilepsy (PSE) in Chinese children.

**Methods:**

Thirty-one children with PSE were screened out of 398 children with epilepsy who were consecutively diagnosed by the video-electroencephalogram (VEEG) monitoring method and by using an intermittent photic stimulation (IPS) test. Their EEGs and clinical features were retrospectively analyzed, and their treatment outcomes were followed up.

**Results:**

PSE accounted for 7.79% (31/398) of children with epilepsy during the observation period in our single epilepsy center. The male to female ratio of PSE was 1:3.43, and the average seizure onset age was 7.8 ± 3.28 years. The highest range of frequency sensitivity of the IPS test for the induction of EEG epileptic discharge or electroclinical seizures was within 10–20 Hz. Electroclinical seizures were induced in 41.94% (13/31) of PSE patients by using the IPS test, while EEG discharge without clinical seizures was induced in 58.06% (18/31) of PSE patients. Among all PSE patients, an IPS-positive reaction in the eye-closure state was induced in 83.87% of patients, and this rate was significantly higher than that in the eye-opened state (41.94%) or eye-closed state (35.48%). (Eye-closure IPS stimulation means: make the subjects close their eyes at the beginning of each stimulation, open their eyes at the end of the stimulation, and close their eyes again at the beginning of the next stimulation, and so on. While Eye-closed IPS stimulation means the stimulation is started after 5 s of eye closure, and the subjects are kept closed throughout the whole process.) The common and effective drugs used for single or combined therapy in PSE children were valproic acid and levetiracetam.

**Conclusion:**

This study provides some useful information about electroclinical characteristics in a cohort of 31 PSE children. It may be beneficial for pediatric neurologists in terms of paying more attention to PSE and correctly dealing with it.

## 1. Introduction

Since the 1940s, bright flickering white light has been considered a reliable method of evoking electroencephalogram (EEG) epileptiform discharge in susceptible patients (1, 2). In 1993, three British TV audiences experienced epileptic seizure when watching a commercial (3). Subsequently, in 1997, 685 Japanese children happened to experience seizures caused by the flashlight stimuli on the screen while watching the vastly popular animated television cartoon series Pocket Monsters (Pokemon). This incident was described as the *Pokemon phenomenon*, which shocked the world (4). Moreover, reports of seizures associated with playing video games were also increasingly produced (5, 6). These events prompted Japan and the United Kingdom to rapidly develop guidelines designed to reduce the risk of seizure during broadcast viewing (7). In fact, such photosensitive epilepsy (PSE) is the most common type of reflex epilepsy (8), which is characterized by photosensitivity, and can be divided into two types: pure PSE and epilepsy with photosensitivity (6, 9). In general, PSE accounts for approximately 10%–20% of children with epilepsy and has a strong genetic background and ethnic differences (10–14). The age of onset of PSE is found predominately around puberty with high incidence in females (15). Although its exact mechanisms are still unclear, studies have shown that some factors may play important roles in the pathogenesis underlying PSE, such as cortical hyperexcitability, abnormal brain structure, abnormal neural functional connectivity, changes in cerebral blood flow, changes in brain energy metabolism, hormone levels, and genetic factors (16, 17). The EEG feature of PSE commonly shows localized or generalized epileptiform discharge induced by intermittent photic stimulation (IPS) or other visual stimuli in daily life, which is also called photoparoxysmal response (PPR) (18, 19). Waltz et al. divided the PPR into grades 1–4, and the PPR related to epilepsy is generally graded 3–4 (20, 21). The prognosis of PSE is generally good, and the photosensitivity of some patients may disappear at the age range of 20–30 (22). However, patients with a family history of PSE may have a relatively poor prognosis. At present, there are few studies on PSE in Chinese children. To fill this research gap, in this study, we retrospectively summarize the electroclinical characteristics in PSE children. To our knowledge, this is the largest sample of PSE in the Chinese population.

## 2. Subject and methods

### 2.1. Study population

In a single center of the Pediatric Neurology Department of the First Hospital of Jilin University, 398 children with epilepsy who were both outpatients and inpatients and completed the IPS test were consecutively recruited from July 2015 to November 2019. The diagnosis of epilepsy was based on the criteria established by the International League Against Epilepsy (ILAE) in 2017. Written informed consents were obtained from all parents and the patients themselves who were more than 8 years of age. Out of the 398 children with epilepsy, 33 who fulfilled the diagnostic criteria of PSE with a positive IPS reaction were screened.

### 2.2. Inclusion and exclusion criteria

The inclusion criteria were as follows: (1) those who met the diagnostic criteria of epilepsy, which was not accompanied by serious mental and motor retardation; (2) those in whom an IPS test, showing a positive result, could be performed. The exclusion criteria were as follows: (1) those having secondary epilepsy with a clear cause; (2) those having epilepsy syndromes with photosensitivity, including the Dravet syndrome, the Lennox–Gastaut syndrome, the West syndrome, and the Doose syndrome; (3) those in whom epilepsy was accompanied by other serious organ diseases; (4) those whose clinical data were defective.

### 2.3. General data evaluation

The general condition, seizures, brain imaging results, EEG results, and the eye status obtained from the IPS test results were analyzed in 31 PSE children who were enrolled in this study. The treatment outcomes of these patients were also followed up.

### 2.4. Fifteen-hour VEEG records and the IPS test

Video EEGs (VEEGs) were performed using the 32-channel Nihon Kohden VEEG-9200 system for 15 h in all 31 PSE children. The photic stimulation frequency ranged from 1 to 60 Hz with a gradient increment of 2 Hz during the IPS test. The results of the EEG and IPS tests were analyzed by two EEG technicians and one clinician separately. The PPR positivity rate was determined in terms of the occurrence of widespread or focal epileptiform discharge induced by IPS. If the EEG discharge was accompanied by visible epileptic seizures during the IPS test, the electroclinical seizures (ECSs) induced by IPS were considered positive. In accordance with what was described in other similar reports, when the IPS test was performed, the patients were asked to keep a safe distance of 30 cm from the stimulating light source, and separate trains of flashes of 10 s duration were used with the following frequencies: 1, 2, 4, 6, 8, 10, 12, 14,16, 18, 20, 25, 30, 40, 50, and 60 Hz. The flash trains were separated by a pause of at least 7 s. IPS was used to determine the lowest (starting at and increasing from 2 Hz) and the highest (starting at and decreasing from 60 Hz) frequencies evoking a generalized grade of 3–4 PPR. These ranges were also assessed in three distinct eye conditions in the same sequence for all 31 patients: eye closure, eyes closed, and eyes open. To discriminate between spontaneous and IPS-evoked discharge, the EEG was initially recorded without IPS for at least 2.5 min with the patients’ eyes open and another 2.5 min with their eyes closed. All tracings were retrospectively reanalyzed. In order to minimize the risk of inducing an epileptic seizure during testing, IPS was terminated once a PPR was induced, which lasted for only 1 s. To establish the range of flashlight frequencies that provoked a PPR, the patients were first exposed to a lower-frequency IPS at a rate that was increased in steps of 2 Hz until a flash frequency was reached, which constantly triggered a PPR within 2 s of IPS. The IPS test was then administered at high frequencies starting from 60 Hz and descending in 2 Hz steps until a flash frequency was reached, which again constantly induced a positive PPR. Thus, the upper and lower limits of the PPR range could be established.

### 2.5. Evaluation criteria of drug efficacy

The efficacy of antiseizure medications (ASMs) can be classified into three types, namely, fully controlled, effective, and ineffective: (1) fully controlled: there is no seizure relapse; (2) effective: seizure frequency is reduced by ≥50% compared with the initial rate; (3) ineffective: seizure frequency is either reduced by <50% or not reduced at all, or even increased.

## 3. Results

### 3.1. General clinical features in the children under study

The 31 PSE children accounted for 7.79% (31/398) of all children with epilepsy within the observation period. Among them, there were 7 males and 24 females; the male to female ratio was 1:3.43. Three patients had a family history of seizures, of which 1 male and 1 female had a clear history of visual-evoked seizures induced while watching TV or playing mobile games. Brain imaging (MRI and CT) was performed in 23 patients, yielding normal results in all.

### 3.2. Age of onset and gender distribution

In our cohort, the onset age ranged from 1 to 13.92 years with an average of 7.84 ± 3.28 years. The highest age of incidence ranged from 6 to 11 years with a proportion of 70.96% (22/31, 2 males and 10 females).

### 3.3. Characteristics of epileptic seizures and VEEG

Among the 31 PSE children, the PSE types, EEG background, EEG interictal discharge, IPS test results, and seizure types during VEEG monitoring were determined, and these are summarized in Table 1.

**Table 1:** Epileptic seizures and EEG characteristics in 31 PSE children.

Characteristics of epileptic seizures and EEG	Number	Proportion (%)
PSE types		
PSE only	1	3.23
Epilepsy with photosensitivity	30	96.77
EEG background		
Normal	30	96.77
Abnormal	1	3.23
Interictal EEG presents epileptiform discharge	28	90.32
Awakening only	1	3.23
Sleep only	2	6.45
Awakening and sleep	25	80.65
No interictal EEG discharge and only positive IPS	3	9.68
IPS-induced PPR	18	58.06
IPS-induced electroclinical seizures	13	41.94
Only IPS-induced seizures	5	16.13
IPS-induced seizures plus spontaneous seizures	8	25.81
No seizures detected during VEEG monitoring	11	35.49
Seizures detected during VEEG monitoring	20	64.51
Absence seizures	7	22.58
Myoclonic seizures	6	19.35
Eyelid myoclonia with or without absence seizures	4	6.45
Absence with atonic seizures	1	3.23
Focal seizures	2	6.45
Eye-closure sensitivity (ECS)	8	25.81

EEG, electroencephalogram; PSE, photosensitive epilepsy; IPS, intermittent photic stimulation; PPR, photo paroxysmal response.

### 3.4. EEG characteristics revealed during the IPS test

The types of IPS-induced EEG discharge in our cohort included extensive discharge, localized discharge, and localized accompanied by extensive discharge. The localized discharge included occipital discharge, temporal discharge, and occipital with other types of parietal discharge. The features of EEG discharge in different eye states are also provided in Table 2. The positivity rate of IPS-induced EEG discharge in the eye-closure state was higher than that in other eye states (*P* < 0.05).

**Table 2:** Characteristics of EEG discharge in different eye states during IPS.

Eye state	Cases	Extensive	Localized	Extensive + Localized
	*n* (%)	*n* (%)	*n* (%)	*n* (%)
Closure	12 (38.71)	9 (29.03)	1 (3.23)	2 (6.45)
Closure + Opened	4 (12.90)	2 (6.45)	0 (0)	2 (6.45)
Closure + Closed	6 (19.35)	3 (9.68)	2 (6.45)	1 (3.23)
Opened	4 (12.90)	1 (3.23)	2 (6.45)	1 (3.23)
Opened + Closed	1 (3.23)	1 (3.23)	0 (0)	0 (0)
Opened + Closure + Closed	4 (12.90)	2 (6.45)	1 (3.23)	1 (3.23)
Total	31 (100)	18 (58.06)	6 (19.35)	7 (22.58)

### 3.5. Photic stimuli frequency range of the PPR

**Figure 1:**
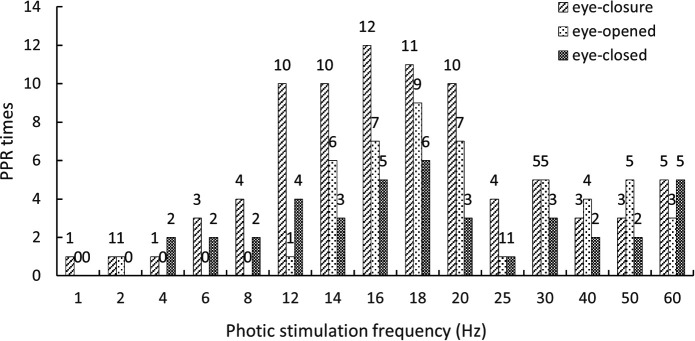
Stimulation frequency distribution of IPS inducing PPR in different eye states.

Among the 31 patients, 2 failed to complete the IPS test because of a rapid induction of the PPR (1 induced PPR in eye closure and 1 in all three eye states mentioned previously); thus, they were excluded from this statistical analysis. A total of 193 PPRs were monitored in the remaining 29 patients. The PPR rate varied with the increasing frequency of photic stimulation during the IPS test, and the maximum frequency sensitivity of the PPR evoked ranged from 10 to 20 Hz (125/193, 64.77%) in all the three eye states of the patients (Figure 1). In the different eye states, 95 PPRs were induced in the eye-closure state, 53 in the eye-opened state, and 45 in the eye-closed state. The occurrence of induced PPR in the eye-closure state was higher than that in the other states. The PPR was recorded in all IPS frequency ranges (1–60 Hz) in the eye-closure state. In other words, it is easier to induce the PPR by IPS in the eye-closure than in the other eye states.

### 3.6. Comparison of electroclinical characteristics in patients with or without ECSs induced by IPS

In our cohort, ECSs were induced by administering the IPS test in 13 patients (41.94%), who were classified as “ES subgroup”. The remaining 18 patients presented a positive PPR (OPPR) without clinical seizures induced during the IPS test, and these patients were classified as “OPPR subgroup”. There was no difference in terms of the male to female ratio, the average onset age of epilepsy, disease duration, and family history between PSE patients with and without clinical seizures induced by IPS (*P* > 0.05).

The types of epileptiform discharge induced by IPS in the ES-subgroup were primarily direct extensive discharge (in 12/13 patients, 92.31%), and one patient presented with extensive discharge secondary to localized discharge. In the OPPR subgroup, six patients (6/18, 33.33%) presented with extensive discharge, localized discharge, and extensive discharge secondary to localized discharge. In general, the extensive discharge in EEG most likely occurs as clinical seizure attacks during the performance of the IPS test (*P* < 0.05).

### 3.7. Electroclinical characteristics of children with epileptiform discharge induced by the IPS test in different eye states

Among the 31 children in our cohort, in 4 (4/31, 12.90%), epileptiform discharge was induced by IPS in all three eye states. Of these four patients, extensive discharge by IPS was induced in two. In the remaining 27 patients, EEG discharge by IPS could not be induced in all the three eye states, while in 11 of them (11/27, 40.74%), ECS was induced. Although the proportion of ECS-induced patients was higher than that of those with EEG discharge in all three eye states, there was no statistical difference between them (*P* > 0.05).

### 3.8. Electroclinical characteristics of children with ECS

In our 31-patient cohort, in eight patients (8/31, 25.81%), EEG discharge was induced by IPS in the eye-closure state, and in five of them (5/8, 62.5%), ECS was induced during the IPS test. In the remaining 23 patients, EEG discharge by IPS was not induced in the eye-closure state, and in 8 of these (8/23, 34.78%), ECS was induced. In the eye-closure state, the ECS rate of the patients in whom EEG discharge was induced by IPS was higher than that of those in whom there was no inducement. However, there was no significant difference between the two subgroups (*P* > 0.05).

### 3.9. ASM treatment response in children with PSE

In our cohort of 31 PSE children, treatment could not be given for 6 patients, and the remaining 25 patients were followed up for 3–55 months, and the median follow-up period was 17 months. There were three patients who never took ASMs, and out of these, two patients were free from seizures, and one patient had intermittent seizures but refused ASM. In the remaining 22 out of the total 25 patients, 20 of them were under ASM monotherapy, which proved efficacious in 19 patients (19/20, 95%). Notably, 17 patients (17/20, 85%) became seizure free. Only in one patient ASM medication was ineffective, and therefore, this patient refused a combination therapy of ASMs. Seizures in the other two patients were completely controlled by using a combination of ASMs. The common ASMs chosen by the clinicians were valproic acid (VPA) and levetiracetam (LEV). The complete control rate of the two drugs was 87.5% (Table 3).

**Table 3:** ASM treatment of 22 children with PSE [*n* (%)].

Medication	Number	Complete control	Effective	Ineffective
Single drug	20	17 (85)	2 (10)	1 (5)
VPA	8	7 (87.5)	1 (12.5)	0 (0)
LEV	8	7 (87.5)	1 (12.5)	0 (0)
OXC	2	2 (100)	0 (0)	0 (0)
LTG	1	1 (100)	0 (0)	0 (0)
TPM	1	0 (0)	0 (0)	1 (100)
Combined	2	2 (100)	0 (0)	0 (0)
LEV + OXC	1	1 (100)	0 (0)	0 (0)
VPA + LEV + OXC	1	1 (100)	0 (0)	0 (0)

ASM, antiseizure medication; PSE, photosensitive epilepsy; LEV, levetiracetam; LTG, lamotrigine; OXC, oxcarbazepine; TPM, topiramate; VPA, valproic acid.

## 4. Discussion

PSE was first described by Gowers in 1885 (23). Since then, technological advances have facilitated easy detection of PSE, and human exposure to photic stimuli has increased because of the growth of new forms of media such as TV and video games. In spite of the fact most epileptic seizures are unprovoked, a subset of patients suffer from reflex epilepsy wherein their seizures are provoked by specific, predictable stimuli. PSE is the most common type of reflex epilepsy. However, there is still a lack of studies on PSE in Chinese children. It is relevant to mention that PPR and PSE are not the same concepts. PPR is more common in children, with incidence rates of 7.6%–8.9%, specifically in adolescent girls when attaining puberty. Moreover, Grade 3–4 PPR responses are significantly more common in children under 10 years of age. Patients with incidental PPR may not have an increased risk for epilepsy (16). Accordingly, 398 children with epilepsy who were subjected to the IPS test were recruited in our hospital. Of these, 31 patients (7.79%) were diagnosed with PSE, with their IPS test results being positive. The number of PSE cases in this study is lower than that in other reports (10%–20%) (15). The reasons for this may be that some children with epilepsy failed to complete the IPS test during the observation period. Moreover, the test results may have been influenced by ethnic differences; however, this point needs to be confirmed by conducting another study with a large sample size.

PSE is a type of age-dependent epilepsy with a preponderant onset of puberty and a predominantly female gender–related condition. The prevalence of PSE in females is 1.5–2 times higher than that in males (24). In our cohort, the average age of onset of PSE children was 7.84 ± 3.28 years, and the male to female ratio was 1:3.43, indicating the possibility of an earlier onset age of PSE, with a higher prevalence in females in Chinese children with epilepsy. This also underscores the influence of sex and steroid hormones such as progestogens, androgens, and estrogens, on seizure susceptibility (25). However, the underlying mechanism is not clear and may be correlated with genetic factors or ethnic differences (26–29).

The types of IPS-induced EEG discharge in our cohort were extensive discharge, localized discharge, and localized with extensive discharge. In the three eye states of the IPS test, the most likely to induce positive reaction is the eye-closure state. Compared with the other eye states, eye closure may promote cerebral cortex excitability more powerfully, which may facilitate a fast and wide spread of photoelectrical signals from the retina (30). In this study, EEG discharge was induced in 83.87% of PSE children in the eye-closure state, which was significantly higher than that in the eye-opened (41.94%) and eye-closed states (35.48%).

The maximum frequency sensitivity of the photic stimulation for PPR induced in the IPS test is considered to range from 8 to 30 Hz (31). Our study indicated that this range was 10–20 Hz. In addition, the PPR was recorded in all IPS frequency ranges (1–60 Hz) in the eye-closure state. The maximum frequency sensitivity of EEG discharge induced by IPS in all the three eye states was also in the range of 10–20 Hz in our PSE cohort, which kept pace with each other.

As for electric seizures, also known as subclinical seizures, EEG presents the characteristics of ictal discharge, but it is not accompanied by symptoms and signs that can be objectively observed or subjectively felt. ECSs mean that EEG presents the characteristics of ictal discharge, which is accompanied by symptoms and signs. In this study, 13 patients (41.94%) presented with ECSs induced by IPS, which was higher than that previously reported (31%) (32). The seizure types of these patients were as follows: myoclonic seizures (4//13, 30.77%), absence seizures (3/13, 23.08%), eyelid myoclonic seizures (2/13, 15.38%), eyelid myoclonic seizures with or without absence (2/13, 15.38%), absence with atonic seizures (1/13, 7.69%), and focal seizures (1/13, 7.69%). The most common seizure type was myoclonic seizures, and this finding is not consistent with that of the reported GTCs in other previous studies (33). However, this still needs to be verified in future studies performed with large samples.

Previous studies have shown that extensive PPR is closely related to clinical seizures (34). In this study, ECSs were induced by IPS in 13 patients (ES subgroup), and the remaining 18 patients tested positive for PPR (OPPR subgroup) induced by IPS. The type of EEG discharge induced by IPS in the ES subgroup was mainly direct extensive discharge (92.31%), and in one patient, there was extensive discharge secondary to localized discharge. Our study showed that extensive EEG discharge was more likely to present as clinical seizure attacks during the performance of the IPS test, which was consistent with the findings of a previous report (34). Therefore, EEG technicians should devise an antiseizure rescue therapy beforehand and pay more attention to the type of extensive discharge when performing the IPS test. It is important to be alert to the possibility of inducing ECSs.

It is considered that EEG discharge induced by IPS in children in all three eye states tends to present as ECSs (35). However, in our cohort, there was no significant difference in terms of the occurrence of ECSs induced by the IPS test between patients with and without EEG discharge in all three eye states (rate: 2/4 vs. 11/27). ECS often coexists with photosensitivity with incidence rates of 20%–36% in photosensitive patients, and it is preponderant in females (36, 37). However, in our cohort, there was no statistical difference in the ECS rate between patients with and without ECSs induced by IPS (rate: 5/8 vs. 8/23), which was inconsistent with the findings in other reports (38). Suspiciously, such discrepancy may be correlated with racial differences or bias resulting from the small sample size of this study.

Generally, PSE has good long-term outcomes, since photosensitivity may disappear at the age of 20–30 years (39). However, PSE patients with a family history of positive rates tend to have a relatively poor prognosis because of the persistent photosensitivity (22). In addition, absence seizures caused by photosensitivity is difficult to relieve with advancing age (40). The treatment determination of PSE varies depending on the severity of seizure attacks. Seizures may be controlled without ASM treatment in a small proportion of pure PSE patients. In our cohort, two patients who did not take ASM medication, never reported seizure recurrence. Accordingly, for PSE children with infrequent seizures, non-drug treatment could be attempted first. Nevertheless, given the complex light environment to which technicians are exposed in modern life, it is very difficult to avoid harmful visual stimulation. In fact, PSE seizures can be induced by various types of visual stimuli. Clinicians need to guide PSE children and their caregivers in taking steps to avoid all kinds of rhythmic or harmful visual stimulation. When harmful photic stimulation is unavoidable, one could avoid seizures by maintaining a monocular vision or wearing special glasses to reduce the total retinal area for receiving light (7, 41, 42).

For the vast majority of patients, ASM treatment is necessary. Occasionally, ASMs must be used in combination with other medications (7). To date, there is a lack of reports on PSE treatment, and especially, studies related to whether ASMs impact PPR are sparse. In this study, positive PPR could be induced by IPS in eight PSE children under ASM therapy and without seizure recurrence. The ASMs included LEV, VPA, oxcarbazepine (OXC), and carbamazepine (CBZ). However, due to the small sample size and irregular follow-up, it was difficult to determine the specific effects of different ASMs on PPR. Customarily, ASM monotherapy is recommended. VPA and lamotrigine (LTG) are the most common drugs for PSE, and VPA is often used as the first line of treatment. If these two drugs are ineffective, LEV, clobazam, and ethosuximide can be considered (7). In our cohort, 25 of the 31 patients were followed up for 3–55 months. Among these 25 patients, 3 never took ASMs. Out of the 22, 20 patients were under ASM monotherapy, and in two, seizures were completely controlled using a combination treatment of ASMs. Among the 20 ASM monotherapy patients, 19 (19/20, 95%) showed effective response to treatment, and seizures were fully controlled especially in 17 patients (17/20, 85%). Only one patient under monotherapy showed ineffective response and refused ASM combination therapy. The common ASMs chosen by the clinicians in our study were VPA and LEV, and the fully controlled rate for both these ASMs was 87.5% (Table 3). Our data suggest that PSE children usually show good response to ASM therapy, and in most of them, seizures can be effectively controlled by a single-drug treatment. It is reported that fewer than 10% of PSE patients are resistant to ASMs, but most of them engage in self-induction or maybe non-compliant to ASMs (7).

It has been reported that gamma oscillations induced by light stimuli assist in the hippocampus CA1 regions promote a reduction of amyloid levels and improve memory in animal models of Alzheimer’s disease (43). Moreover, there are also a few clinical reports to the effect that light stimulation can reduce epileptic seizures (44–46). Conversely, it is well known that rhythmic light stimuli can induce seizures. In this context, there arises a question. Can a light stimulation mode with appropriate parameters disrupt seizure initiation, and, in turn, reduce the abnormal potentiation in epileptic circuits? Reverse thinking, the light stimulation with parameters that opposite to that easy to trigger epileptic seizures may be potentially worth studied in future research to control epileptic seizures. Thus, a non-invasive and non-pharmacological treatment of therapeutic light for epilepsy may be developed, which hopefully, will become a useful approach for epilepsy therapy in the future.

However, there are some limitations in this study. First, because of the fact that some epileptic patients could not complete the IPS test, our cohort is inherently defective to represent epilepsy patients as a whole in our single center within a specified period of time; thus some element of bias on reporting the incidence of PSE in children could have crept into this study. Second, because of the small sample size, the overall clinical characteristics of PSE in Chinese children cannot be captured in all its dimensions. Third, because of the retrospective nature of the study, some children with PSE could not be re-examined by using the IPS test and be followed up regularly; also, the impact of ASMs on PPR remains to be further investigated. In addition, it should be emphasized that due to the exclusion of children with photosensitive intractable epilepsy syndrome (such as the Dravet syndrome), a more objective understanding of the response of such children to ASM treatment may be lacking in this study. Photosensitivity may also be one of the possible mechanisms underlying ASM resistance in children with intractable epilepsy syndrome, and therefore, this aspect can be examined in a future study.

## Data Availability

The original contributions presented in the study are included in the article/Supplementary Material, and further inquiries can be directed to the corresponding author.
